# Persistent impact of antenatal maternal anaemia on child brain structure at 6–7 years of age: a South African child health study

**DOI:** 10.1186/s12916-024-03838-6

**Published:** 2025-02-21

**Authors:** Jessica E. Ringshaw, Chanelle J. Hendrikse, Catherine J. Wedderburn, Layla E. Bradford, Simone R. Williams, Charmaine N. Nyakonda, Sivenesi Subramoney, Marilyn T. Lake, Tiffany Burd, Nadia Hoffman, Annerine Roos, Katherine L. Narr, Shantanu H. Joshi, Steven C. R. Williams, Heather J. Zar, Dan J. Stein, Kirsten A. Donald

**Affiliations:** 1 Department of Paediatrics and Child Health, Red Cross War Memorial Children’s Hospital, University of Cape Town, Cape Town, South Africa; 2 Neuroscience Institute, University of Cape Town, Cape Town, South Africa; 3 Centre for Neuroimaging Sciences, Department of Neuroimaging, Kings College London, London, UK; 4 Department of Clinical Research, London School of Hygiene & Tropical Medicine, London, UK; 5 South African Medical Research Council (SAMRC), Unit on Child & Adolescent Health, University of Cape Town, Cape Town, South Africa; 6 Department of Psychiatry & Mental Health, University of Cape Town, Cape Town, South Africa; 7 South African Medical Research Council (SAMRC), Unit on Risk and Resilience in Mental Disorders, University of Cape Town, Cape Town, South Africa; 8 Department of Neurology, University of California Los Angeles, Los Angeles, USA; 9 Department of Psychiatry and Biobehavioural Sciences, University of California Los Angeles, Los Angeles, USA; 10 Department of Bioengineering, University of California Los Angeles, Los Angeles, USA

**Keywords:** Neuroimaging, Magnetic resonance imaging, Neurodevelopment, Antenatal maternal anaemia, Child brain structure

## Abstract

**Background:**

This study aimed to determine whether associations of antenatal maternal anaemia with smaller corpus callosum, caudate nucleus, and putamen volumes previously described in children at age 2–3 years persisted to age 6–7 years in the Drakenstein Child Health Study (DCHS).

**Methods:**

This neuroimaging sub-study was nested within the DCHS, a South African population-based birth cohort. Pregnant women were enrolled (2012–2015) and mother–child dyads were followed prospectively. A sub-group of children had magnetic resonance imaging at 6–7 years of age (2018–2022). Mothers had haemoglobin measurements during pregnancy and a proportion of children were tested postnatally. Maternal anaemia (haemoglobin < 11 g/dL) and child anaemia were classified using WHO and local guidelines. Linear modeling was used to investigate associations between antenatal maternal anaemia status, maternal haemoglobin concentrations, and regional child brain volumes. Models included potential confounders and were conducted with and without child anaemia to assess the relative roles of antenatal versus postnatal anaemia.

**Results:**

Overall, 157 children (*Mean* [*SD*] age of 75.54 [4.77] months; 84 [53.50%] male) were born to mothers with antenatal haemoglobin data. The prevalence of maternal anaemia during pregnancy was 31.85% (50/157). In adjusted models, maternal anaemia status was associated with smaller volumes of the total corpus callosum (adjusted percentage difference, − 6.77%; *p* = 0.003), left caudate nucleus (adjusted percentage difference, − 5.98%, *p* = 0.005), and right caudate nucleus (adjusted percentage difference, − 6.12%; *p* = 0.003). Continuous maternal haemoglobin was positively associated with total corpus callosum (*β* = 0.239 [CI 0.10 to 0.38]; *p* < 0.001) and caudate nucleus (*β* = 0.165 [CI 0.02 to 0.31]; *p* = 0.027) volumes. In a sub-group (*n* = 89) with child haemoglobin data (*Mean* [*SD*] age of 76.06 [4.84]), the prevalence of antenatal maternal anaemia and postnatal child anaemia was 38.20% (34/89) and 47.19% (42/89), respectively. There was no association between maternal and child anaemia (*χ*^2^ = 0.799; *p* = 0.372), and child anaemia did not contribute to regional brain volume differences associated with maternal anaemia.

**Conclusions:**

Associations between maternal anaemia and regional child brain volumes previously reported at 2–3 years of age were consistent and persisted to 6–7 years of age. Findings support the importance of optimising antenatal maternal health and reinforce these brain regions as a future research focus.

**Supplementary Information:**

The online version contains supplementary material available at 10.1186/s12916-024-03838-6.

## Background

From conception through early postnatal life, the developing child brain is extremely sensitive to environmental influences [1–3]. While the importance of this first 1000-day window is well-known, recent research is providing increasing evidence to support a renewed focus on optimising antenatal maternal well-being for improved neurodevelopmental outcomes in children [1]. In recognising the foetal origins of brain health and the persistent impact of early risk, maternal anaemia during pregnancy is an important health priority for targeted prevention and intervention strategies [4].

Anaemia, indicated by low serum haemoglobin, affects one quarter of the world’s population [5]. Women and children bear the greatest burden with 30% of women of reproductive age and 36% of pregnant women estimated to be anaemic [6]. In considering possible aetiologies for anaemia, chronic iron deficiency has consistently been identified as the most common underlying direct cause [5], accounting for approximately 50% of cases in this group [6]. Although anaemia is a global health priority, it is most prevalent in low- and middle-income countries (LMICs), particularly in Africa and South Asia [6]. This is due to multiple risk factors including malnutrition, food insecurity, and infectious disease [7–9], all of which are highly prevalent in these regions [10–13]. Given that progress in reducing anaemia has stagnated between 2000 and 2019 [6], it has been identified by the World Health Organization (WHO) as a Sustainable Development Goal (SDG) for accelerated action [5].

The risk of anaemia rises during pregnancy due to increased blood volume to support the haemoglobin-facilitated supply of oxygen to the foetus, higher metabolic demand of the developing brain, and foetal iron loading [2, 14]. In turn, antenatal maternal anaemia and iron deficiency have consistently been associated with a greater probability of poor maternal and infant health outcomes including placental abruption, maternal shock, ICU admission, maternal mortality, post-partum haemorrhage, foetal growth restriction, stillbirth, prematurity, and low birthweight [15–17]. Additionally, antenatal maternal anaemia and iron status are well-known risk factors for poorer general paediatric neurocognitive outcomes in multiple settings [18–20], including South Africa [1]. Similarly, an association has been identified between maternal anaemia during early pregnancy and increased risk of a range of neurodevelopmental conditions including autism spectrum disorder (ASD), attention-deficit hyperactivity disorder (ADHD), and intellectual disability (ID) [21].

Non-invasive approaches such as neuroimaging provide an opportunity to objectively explore the potential neurobiological impact of anaemia on the developing brain comparably across cultures and settings [22, 23]. However, while recent neuroimaging research using magnetic resonance imaging (MRI) suggests a striking neural vulnerability to iron deficiency and anaemia [24–29], this remains an emergent field with varying research foci. For example, existing studies on brain imaging outcomes associated with postnatal foetal (cord blood) or child (venous blood) iron and anaemia status do not address the role of maternal anaemia [26, 27], and emphasise severe pathological presentations due to haemorrhage [26]. Other neuroimaging research has been designed with brain imaging measures as the exposure variable to assess postnatal brain iron concentration as a risk factor for poor cognitive outcomes [28] or developmental disorders in childhood [29]. The few studies that have investigated maternal iron deficiency and anaemia exposure have targeted particular brain structures [24], used different MRI modalities (structural [24] versus diffusion tensor imaging [25]), and were limited by the use of self-report indicators of maternal iron based on dietary intake [25]. They also largely focused on neonatal brain outcomes, resulting in very little being known about the relative effects of antenatal versus postnatal anaemia and the persistence of effects in early and middle childhood [24, 25].

Given the scarcity of MRI studies in LMICs [30, 31] and the particularly high prevalence of anaemia in Africa [6], a neuroimaging sub-study of the Drakenstein Child Health Study (DCHS; South African birth cohort) [32, 33] investigated the associations between antenatal maternal anaemia, postnatal child anaemia, and child brain structure at 2–3 years of age [4]. In this cohort, maternal anaemia during pregnancy was associated with smaller volumes of the total corpus callosum, bilateral caudate nuclei, and left putamen in the child brain. The corpus callosum is the primary commissural white matter tract connecting the two brain hemispheres [34] and the iron-rich basal ganglia [35, 36] are important components of the limbic and motor systems [35–39]. Volumetric changes in these brain structures are of clinical relevance given their key role in multiple neurocognitive domains including executive functioning, motor skills, learning and memory, socio-emotional regulation, and visuospatial intelligence [28, 34, 39–44].

The DCHS neuroimaging study on toddlers highlights a critical need to optimise the timing of anaemia interventions. However, further research is necessary to corroborate results and to determine whether these novel neuroimaging findings persist with age. This may aid in understanding the longitudinal impact of maternal risk on patterns of child brain development by identifying whether structural brain changes are long-lasting or indicative of a temporary delay in growth. The aim of this study was to determine whether the association between antenatal maternal anaemia and smaller corpus callosum and basal ganglia volumes described in toddlers (aged 2–3 years) from the DCHS persisted in this birth cohort at school age (6–7 years of age).

## Methods

### Study design and setting

The DCHS is an observational population-based birth cohort located in the peri-urban Drakenstein district of Cape Town, South Africa [32, 33]. This community is characterised by low socio-economic status (SES) and multiple health and psychosocial adversities with a high prevalence of risk factors such as maternal HIV, food insecurity, and malnutrition. However, more than 90% of the population have access to public health services, with TC Newman and Mbekweni Clinics being the two primary healthcare centres for this study. Pregnant women were recruited for the DCHS while attending antenatal clinic visits, and well-characterised mother–child dyads have been followed prospectively.

### Participants

Between 2012 and 2015, 1125 pregnant women were enrolled in the DCHS and 1143 live births were included with good retention in postnatal follow-up care. In a nested neuroimaging study [45], a sub-group of children were invited for MRI as neonates (2012–2016), at 2–3 years (2015–2018), and at 6–7 years (2018–2022). Exclusion criteria included (1) medical comorbidity (genetic syndrome, neurological disorder, or congenital abnormality); (2) gestation less than 36 weeks; (3) low Apgar score (less than 7 at 5 min); (4) neonatal intensive care admission; (5) maternal use of illicit drugs; (6) MRI contraindications; and (7) child HIV infection. Of the eligible children, those who were scanned at birth were followed up at subsequent imaging sessions alongside additional children who were selected at the 2–3-year timepoint based on known neurodevelopmental risk factors (maternal HIV and alcohol exposure) and a randomly selected control group. Previous DCHS research has demonstrated comparability between the nested neuroimaging sub-study sample and the full cohort [45].

Findings regarding the association between antenatal maternal anaemia and child brain structure at 2–3 years of age in this cohort have been published [4]. The current study is focused on the 6–7-year timepoint, at which 157 children had both useable structural neuroimaging data (see “ Neuroimaging outcomes” section below) and maternal haemoglobin data for inclusion in analysis. A study flowchart is available as supplementary information (Additional File 1: Figure S1) [4, 32, 46].

### Measures

#### Contextual measures

Demographic, environmental, psychosocial, and clinical data for mother–child dyads were collected antenatally and postnatally for descriptive purposes and for consideration as covariates. All mothers underwent repeat testing for HIV during pregnancy and infants were tested postnatally as per national guidelines. Birth details were obtained by study staff at delivery and child gestational age was calculated using ultrasonography in the second trimester of pregnancy or, where this was unavailable, symphysis-fundal height or maternal report of the most recent menstrual cycle. Child anthropometry measures were observed and classified according to WHO guidelines [47] at routine study visits as well as neuroimaging sessions. Maternal tobacco smoking in pregnancy was self-reported and a dichotomous classification of antenatal alcohol use was retrospectively assessed using the Alcohol, Smoking, and Substance Involvement Screening Test (ASSIST) [48].

#### Anaemia and iron deficiency

Antenatal maternal and postnatal child anaemia was assessed based on serum haemoglobin measurements in pregnancy and childhood, respectively. Maternal haemoglobin measurements were acquired using rapid tests at antenatal clinic visits as per standard healthcare policy, and iron and folic acid supplementation was recommended as per national guidelines. This data was abstracted from clinical records by trained DCHS staff at study enrolment. Where multiple haemoglobin measurements were available, the lowest value was selected to represent the most severe maternal anaemia presentation. Based on WHO guidelines [49], haemoglobin levels of < 11 g/dL during pregnancy were used to classify pregnant women as anaemic. Further classifications into mild (haemoglobin 10.0–10.9 g/dL), moderate (haemoglobin 7.0–9.9 g/dL), and severe anaemia (haemoglobin < 7.0 g/dL) were determined. Child haemoglobin was only available for children who presented at hospital with lower respiratory tract infections between birth and the MRI scan, as part of a full blood count. Child anaemia was classified based on age-specific cut-offs using the WHO guidelines for all measurements in children aged over 6 months and local guidelines (Groote Schuur Hospital/University of Cape Town Pathology Laboratory) for children under 6 months (Additional File 1: Table S1). A dichotomous variable for child anaemia status was created based on meeting anaemia criteria at least once between birth and 6–7 years of age.

#### Neuroimaging outcomes

Based on earlier findings [4], structural MRI was chosen as the most relevant imaging measure. Brain volume was acquired on a 3-Tesla Siemens Skyra MRI system at the Cape Universities Body Imaging Centre (CUBIC) using a 32-channel head-coil. All children were scanned awake while watching a movie inside the MRI machine. The neuroimaging protocol and MRI specifications are available as supplementary material (Additional File 1: Text S1) [45, 46].

All structural brain scans were processed with FreeSurfer Version 7.1.1 using an automated process of cortical reconstruction and volumetric segmentation [50, 51]. Regional brain volumes ($${mm}^{3})$$ were extracted for analysis using the Desikan-Killiany atlas [52] and an inbuilt probabilistic atlas [50] for cortical parcellation and subcortical segmentation, respectively [52]. Given that antenatal maternal anaemia was associated with smaller corpus callosum, caudate nucleus, and putamen volumes in the DCHS analysis at 2–3 years of age [4], these brain regions were chosen as key a priori regions of interest (ROIs) for a targeted analysis at 6–7 years of age. However, based on broader literature [24–26, 28] and the identification of iron-rich brain structures, other potentially vulnerable brain regions including the cerebellum and subcortical regions such as the hippocampus, amygdala, thalamus, nucleus accumbens, and pallidum were identified for exploratory analyses. All subcortical structures, including the caudate nucleus and putamen, were segmented into left and right hemispheres. The corpus callosum was segmented into posterior, mid-posterior, central, mid-anterior, and anterior regions. The total corpus callosum volume was computed by summing all individual sub-regions, and the body was defined as the sum of the mid-posterior, central, and mid-anterior volumes. Left and right hemispheres of the cerebellum were further divided into the cerebellar white matter and cerebellar cortex. Global volumes such as the total cerebral cortex, total grey matter, and total cerebral white matter were also investigated in exploratory analyses. Intracranial volume (ICV) was included as a covariate in analyses to account for normal inter-individual variability in brain size.

All regional segmented output (*n* = 204) was subject to a standardised quality control check using the ENIGMA Cortical Quality Control Protocol 2.0 [46]. This was conducted independently by two senior research staff with experience in neuroimaging processing and analysis. Subjects with consistent failures across all brain regions on internal and external quality control (QC) were excluded (*n* = 36). Further decisions on inclusion in the dataset were made by identifying participants that emerged as statistical outliers in SPSS (using Tukey’s method) [53] for subcortical (*n* = 0) and corpus callosum (*n* = 1) ROIs. Overall, a sample of 167 participants passed the visual and statistical QC testing, of which 157 had maternal haemoglobin data (Additional File 1: Figure S1) [4, 32, 46].

### Statistical analysis

#### Sample characteristics

Demographic data and clinical characteristics were presented as means and standard deviations for continuous variables and frequencies for categorical variables. Sociodemographic and clinical (e.g. maternal exposures) group differences between children with antenatal maternal anaemia exposure and children without antenatal maternal anaemia exposure were calculated using unpaired *t*-tests for continuous data and chi-squared tests or Fisher’s exact tests for categorical data.

#### Maternal anaemia status

The exposure variable was antenatal maternal anaemia status (dichotomised as anaemic versus non-anaemic based on WHO haemoglobin cut-offs for pregnancy) and the outcomes were regional child brain volumes selected a priori. Between-group differences were investigated using multivariate analysis of variance (MANOVA) general linear models (GLMs). Given that this analysis aimed to determine whether previously identified findings in 2–3-year-old children persist with age at 6–7 years in the same cohort, a similar statistical approach was conducted. This included a targeted analysis for key a priori ROIs, namely the corpus callosum, caudate nucleus, and putamen. However, separate MANOVAs for exploratory analyses of other subcortical regions, the cerebellum, and global volumes were run to ensure no emerging findings were missed.

A separate set of MANOVA models were performed for grey (left and right hemispheric volumes of subcortical structures) and white (individual corpus callosum regions) matter ROIs. Models were built using a hierarchical stepwise approach with (1) an unadjusted model assessing group differences without the inclusion of covariates, (2) a partially adjusted model including age at scan, sex, SES (represented by maternal education and total household income), and ICV as covariates known to affect brain volume a priori [1, 54], and (3) a fully adjusted model including maternal exposures with demonstrated group differences, placing a particular focus on antenatal alcohol exposure which has consistently been associated with smaller corpus callosum volumes in the broader literature [55] and is known to interact with iron metabolism at a physiological level [56, 57]. A series of fully adjusted post hoc univariate analyses (ANOVAs) were additionally performed for each individual ROI, correcting for multiple comparisons using the false discovery rate (FDR) method [58]. Separate ANOVAs were conducted to assess the association between antenatal maternal anaemia status and overall summed volumes for the body and total corpus callosum.

In comparing volumes based on maternal anaemia status, adjusted mean differences were calculated using pairwise comparisons of estimated marginal means based on the fully adjusted MANOVA and ANOVA models. Percentage differences were calculated using the adjusted mean difference relative to the unadjusted mean volume in the control group (no maternal anaemia) for each brain structure.

#### Maternal haemoglobin concentration

In regions where an association between antenatal maternal anaemia status and child brain volumes was observed (*p* < 0.05), we explored hierarchical multivariable linear regression models using standardised regression coefficients (*β*) for continuous maternal haemoglobin concentrations. This allowed us to assess the relationship between maternal anaemia severity and regional child brain volumes.

#### Child anaemia sub-analysis

To explore the relative role of postnatal child anaemia on regional child brain volumes in a sub-analysis (children with both maternal and child haemoglobin data), child anaemia status was included as an additional covariate for consideration in the established fully adjusted models for maternal anaemia status described above.

#### Sensitivity analyses and statistical considerations

Sensitivity analyses were conducted to consider the potential role of a broader range of factors, including timing. Firstly, we adjusted for trimester of pregnancy given the anticipated increase in maternal blood volume and haemoglobin with gestational time [49]. Secondly, other relevant clinical maternal exposures such as HIV and smoking, both of which are prevalent in this community, were adjusted for in the models to account for any unmeasured confounding [59]. Lastly, tobacco is known to increase haemoglobin concentrations resulting in the underestimation of functional anaemia in smokers [60]. Therefore, all models were also replicated with an adjusted measure of haemoglobin after correction (Hb—0.3 g/dL) and anaemia status as per the WHO guidelines.

All analyses were conducted using SPSS. A two-sided significance level of *p* < 0.05 was used throughout. Collinearity and biological plausibility was considered in the establishment of all models and checks for assumptions including normality of residuals and homogeneity of variance were conducted throughout.

## Results

### Sample characteristics

In this neuroimaging sample (see Table 1), a total of 157 children between 69 and 90 months of age (*Mean* [*SD*] age of 75.54 [4.77]; 84 [53.50%] male) were born to mothers with antenatal haemoglobin data. Maternal haemoglobin was measured during pregnancy, predominantly in the first (74/157 [47.13%]) and second trimester (81/157 [51.59%]), at a median (IQR) of 14 (7–19) weeks. Overall, 50/157 (31.85%) of the mothers were found to have been anaemic (WHO; haemoglobin < 11 g/dL) during pregnancy, of which 27/50 (54%) had mild anaemia (10.0 to 10.9 g/dL) and 23/50 (46%) had moderate anaemia (7.0 to 9.9 g/dL). None of the mothers had severe anaemia (haemoglobin < 7 g/dL) during pregnancy.

**Table 1:** Sample characteristics of children born to mothers with and without anaemia during pregnancy (*n* = 157)

Variable^a^	Total sample (*n* = 157)		
	Maternal anaemia (*n* = 50)	No maternal anaemia (*n* = 107)	*p*
**Pregnancy characteristics**			
Anaemia status in pregnancy^b^			
Mild	27 (54.00)	n/a	n/a
Moderate	23 (46.00)	n/a	n/a
Severe	0	n/a	n/a
Maternal Hb during pregnancy (g/dL)^e^	9.82 (0.71)	12.25 (0.95)	< 0.001***
Trimester of pregnancy Hb measured^c, f^			
First	14 (28.00)	60 (56.07)	0.002**
Second	35 (70.00)	46 (42.99)	
Third	1 (2.00)	1 (0.93)	
**Maternal characteristics**			
Monthly household income (ZAR)			
< 1000	17 (34.00)	28 (26.17)	0.363
1000–5000	29 (58.00)	63 (58.88)	
> 5000	4 (8.00)	16 (14.95)	
Education^f^			
Primary	5 (10.00)	6 (5.61)	0.771
Some secondary	26 (52.00)	57 (53.27)	
Completed secondary	17 (34.00)	40 (37.38)	
Tertiary	2 (4.00)	4 (3.74)	
Employed	11 (22.00)	38 (35.51)	0.089
Age at delivery (years)	27.28 (6.34)	27.93 (5.56)	0.512
Smoking during pregnancy	18 (36.00)	37 (34.58)	0.862
Alcohol during pregnancy	22 (44.00)	26 (24.30)	0.013*
HIV infection during pregnancy	19 (38.00)	30 (28.04)	0.209
Weight 6 weeks postpartum (kg)^g^	66.34 (18.14)	70.75 (15.56)	0.184
BMI 6 weeks postpartum (kg)^g^	26.55 (6.62)	27.99 (5.68)	0.236
**Child characteristics**			
Age at scan (months)	75.14 (4.81)	75.73 (4.76)	0.473
Sex (boys)	26 (52.00)	58 (54.21)	0.796
HIV infection	0	0	n/a
Gestational age at birth (weeks)	38.68 (2.54)	38.92 (2.00)	0.530
Birth weight (g)^d^	3077.60 (642.26)	3074.32 (586.60)	0.975
Birth length (cm)^d^	49.32 (3.88)	49.44 (3.80)	0.861
Birth head circumference (cm)^d^	33.61 (2.38)	33.51 (1.92)	0.782
WAZ at 6 years^d,g^	− 0.46 (1.18)	− 0.22 (1.02)	0.202
HAZ at 6 years^d,g^	− 0.5 (1.12)	− 0.28 (1.02)	0.228
BMIZ at 6 years^d,g^	− 0.26 (0.95)	− 0.09 (1.04)	0.338
**Child neuroanatomical variables**			
Intracranial volume (mm^3^)	1,328,880.40 (123,752.08)	1,305,553.07 (152,614.64)	0.346

Abbreviations: Hb, haemoglobin; BMI, body mass index (calculated as weight in kilograms divided by height in meters squared); g, grams; HAZ, *z*-scores for height-for-age; BMIZ, *z*-scores for BMI-for-age; WAZ, *z*-scores for weight-for-age; ZAR, South African Rand. SI conversion factor: To convert to haemoglobin grams per litre, multiply by 10

^a^Values for continuous variables are presented as: mean ± standard deviation (range). Values for categorical variables are presented as: number (%)

^b^Maternal anaemia during pregnancy was classified according to the WHO threshold of Hb<11g/dL. Severity classifications were defined as mild (10.0–10.9g/dL), moderate (7.0–9.9g/dL), and severe (<7.0g/dL)

^c^Trimester of pregnancy defined as first (0–12 weeks), second (13–27 weeks), and third (28 weeks onwards)

^d^The birth anthropometric measurements were conducted by trained labour staff in the ward. Infant length was measures in cm to the nearest completed 0.5cm and weight was measured in kg (and converted to grams). Child weight and length measurements at 6–7 years of age were converted to *z*-scores based on age and sex using Anthro software for WAZ, HAZ, and HCZ. Children were classified as underweight, stunted, or having microcephaly if they had *z*-scores of less than −2 SDs

^e^Levene’s test was significant. *T*-test results were interpreted based on equal variance not assumed

^f^Fisher’s exact test result interpreted due to one or more cells having an expected count of less than 5

^g^Missing values: maternal weight 6 weeks postpartum (*n*=40), maternal BMI 6 weeks postpartum (*n*=41), WAZ at 6 years (*n*=2), HAZ at 6 years (*n*=2), BMIZ at 6 years (*n*=3)

*p is significant at <0.05, ** *p* is significant at <0.01, ***p is significant at <0.001

There were no group differences in maternal or child sociodemographic characteristics and anthropometric measures. While antenatal maternal smoking and maternal HIV infection were prevalent across the whole neuroimaging sample, there were no group differences in these exposures. However, antenatal maternal alcohol use was significantly more prevalent in mothers with anaemia during pregnancy (22/50 [44%]) than mothers without anaemia during pregnancy (26/107 [24%]).

### Antenatal maternal anaemia status and child brain structure

#### Corpus callosum

**Fig. 1:**
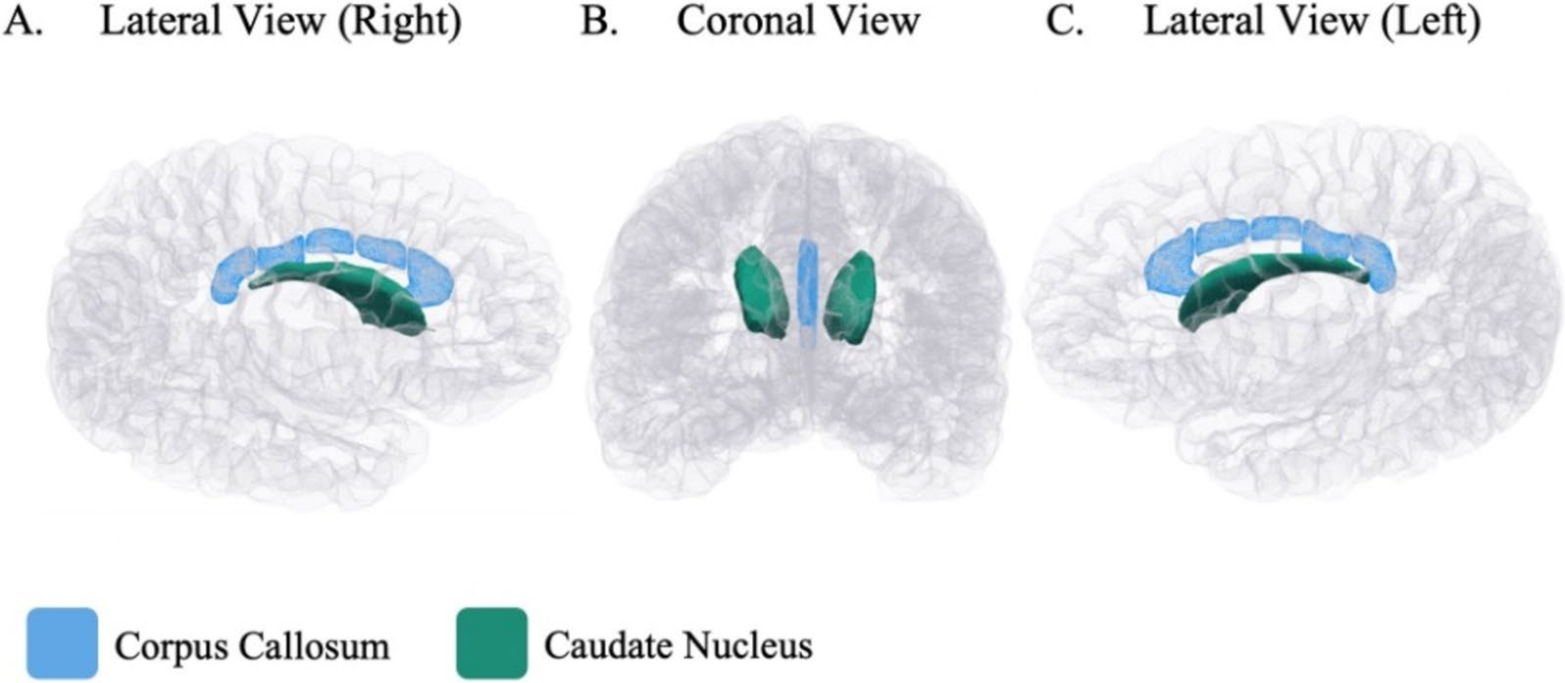
Regional child brain volumes at 6–7 years associated with antenatal maternal anaemia visualised on a cortical surface. Glass brain image of regional child brain volumes visualised on a cortical surface from lateral views (right and left hemispheres of the brain) and a coronal view. In children aged 6–7 years, the brain structures associated with antenatal maternal anaemia were the total corpus callosum (blue) and total caudate nucleus (green)

In a partially adjusted MANOVA model, antenatal maternal anaemia had a main effect on the corpus callosum regions overall, *F*(5,146) = 2.719, *p* = 0.022. This effect was no longer significant in the fully adjusted model which included antenatal alcohol exposure, *F*(5,145) = 2.195, *p* = 0.058. However, fully adjusted post hoc univariate analyses (see Table 2) revealed that children born to mothers who were anaemic during pregnancy had significantly smaller volumes of the posterior (*p* = 0.017), mid-posterior (*p* = 0.009), and central (*p* = 0.018) regions than children born to mothers who were not anaemic during pregnancy, after multiple comparisons correction. Following on from these findings, in fully adjusted ANOVAs, the volumes of the body and the total corpus callosum were found to be smaller in children born to mothers with maternal anaemia during pregnancy (Body: *M* = 1221.09 mm^3^, *SD* = 234.86; Total: *M* = 2682.47 mm^3^, *SD* = 444.82) than children born to mothers without maternal anaemia during pregnancy (Body: *M* = 1322.73 mm^3^, *SD* = 220.45; Total: *M* = 2876.52 mm^3^, *SD* = 387.64), *p* = 0.005 and *p* = 0.003). In considering adjusted mean differences, this corresponds with smaller volumes of approximately 7.89% and 6.77%, respectively. These associations between antenatal maternal anaemia and the corpus callosum (see Fig. 1) had medium effect sizes for the body (*partial η*^*2*^ = 0.053) and total (*partial η*^*2*^ = 0.059) volumes.

**Table 2:** Effects of antenatal maternal anaemia on regional child brain volumes of interest with and without adjusting for covariates (*n* = 157)

Brain region	Mean volume (mm^3^)				Maternal anaemia status					
					Unadjusted GLM^1^		Partially adjusted GLM^2^		Fully adjusted GLM^3^	
	No maternal anaemia Mean (SD) (*n* = 107)	Maternal anaemia Mean (SD) (*n* = 50)	Adjusted mean volume difference (95% CI)^f^	Adjusted mean volume difference (%)^f^	*p*^d^	Partial *η*^2^ (95%CI)	*p*^d^	Partial *η*^2^ (95% CI)	*p*^d^	Partial *η*^2^ (95% CI)
**Corpus callosum**										
Posterior^a^	725.27 (107.11)	674.27 (138.77)	− 49.24 (− 89.64 to − 8.83)	− 6.79	0.013*^e^	0.039	0.010**^e^	0.044	0.017**^e^	0.037
Mid-Posterior^a^	381.39 (80.73)	341.90 (83.02)	− 35.49 (− 62.09 to − 8.88)	− 9.31	0.005**^e^	0.049	0.003**^e^	0.056	0.009**^e^	0.045
Central^a^	482.24 (99.33)	445.59 (89.15)	− 39.29 (− 71.77 to − 6.82)	− 8.15	0.028*^e^	0.031	0.011**^e^	0.042	0.018*^e^	0.037
Mid-Anterior^a^	459.10 (101.23)	433.60 (100.80)	− 29.58 (− 62.43 to 3.27)	− 6.44	0.143	0.014	0.067	0.022	0.077	0.021
Anterior^a^	828.52 (132.61)	787.10 (122.96)	− 41.06 (− 80.96 to − 1.15)	− 4.96	0.064	0.022	0.017*^e^	0.038	0.044*	0.027
Body^c^	1322.73 (220.45)	1221.09 (234.86)	− 104.36 (− 176.06 to − 32.66)	− 7.89	0.009**	0.043	0.002**	0.061	0.005**	0.053
Total^c^	2876.52 (387.64)	2682.47 (444.82)	− 194.65 (− 321.06 to − 68.25)	− 6.77	0.006**	0.048	< 0.001***	0.070	0.003**	0.059
**Subcortical regions**										
Caudate Nucleus										
Left^b^	3748.31 (505.90)	3551.52 (442.70)	− 224.18 (− 378.51 to − 69.85)	− 5.98	0.020*^e^	0.035	0.005*^e^	0.052	0.005**^e^	0.052
Right^b^	3944.82 (531.17)	3740.32 (439.08)	− 241.48 (− 400.48 to − 82.49)	− 6.12	0.019*^e^	0.035	0.004*^e^	0.055	0.003**^e^	0.057
Putamen										
Left^b^	5149.95 (554.81)	5048.92 (598.77)	− 109.06 (− 284.50 to 66.38)	− 2.12	0.302	0.007	0.194	0.011	0.221	0.010
Right^b^	5176.46 (543.71)	5114.14 (578.16)	− 78.26 (− 245.75 to 89.22)	− 1.51	0.513	0.003	0.360	0.006	0.357	0.006

Corpus callosum body: Sum of mid-posterior, central, and mid-anterior regions. Total Corpus callosum: Sum of posterior, mid-posterior, central, mid-anterior, and anterior regions

^1^Unadjusted model including only antenatal maternal anaemia status

^2^Partially adjusted model including antenatal maternal anaemia status, ICV, child age and sex at scan, SES (indicated by maternal education and household income)

^3^ Fully adjusted model including antenatal maternal anaemia status, ICV, child age and sex at scan, SES (indicated by maternal education and household income), and antenatal alcohol exposure

^a,b^Fully adjusted MANOVA models run separately for the corpus callosum and caudate nucleus regions

^c^Fully adjusted ANOVA models run separately on the body and total corpus callosum summed volumes

^d^*p* values are adjusted for covariates but are not corrected for multiple comparisons

^e^Survived FDR correction for multiple comparisons. For the body and total corpus callosum where univariate analyses (ANOVAs) were run, multiple comparisons was not applicable

^f^The adjusted mean difference was calculated from the fully adjusted MANOVA models via post-hoc pairwise comparison using estimated marginal means. A negative mean difference and the corresponding percentage difference represent a smaller volume in children born to mothers with anaemia during pregnancy

*p is significant at < 0.05, **p is significant at < 0.01, ***p is significant at < 0.001

#### Caudate nucleus and putamen

In a fully adjusted MANOVA model, antenatal maternal anaemia had a significant main effect on the subcortical regions of interest, *F*(4,146) = 2.458, *p* = 0.048. Post hoc univariate ANOVAs (see Table 2) revealed smaller volumes of the left and right caudate nucleus in children born to mothers with maternal anaemia during pregnancy (Left: *M* = 3551.52 mm^3^, *SD* = 442.70; Right: *M* = 3740.32 mm^3^, *SD* = 439.08) compared to those whose mothers were not anaemic during pregnancy (Left: *M* = 3748.31 mm^3^, *SD* = 505.90; Right: *M* = 3944.82 mm^3^, *SD* = 531.17), *p* = 0.005 and *p* = 0.003, respectively. In considering adjusted mean differences, this corresponds with smaller volumes of approximately 5.98% and 6.12%, respectively. This association between antenatal maternal anaemia and the caudate nucleus (see Fig. 1) demonstrated a medium effect size for both the left (*partial η*^*2*^ = 0.052) and right (*partial η*^*2*^ = 0.057) hemispheres. It is noted that the sequential adjustment for antenatal alcohol exposure in the fully adjusted model did not change the effects of antenatal maternal anaemia on the caudate nucleus. There was no significant effect of antenatal maternal anaemia on the left or right putamen in this age group.

#### Exploratory analyses

In an exploratory analysis based on the fully adjusted model, there were no other subcortical regions associated with antenatal maternal anaemia (Additional File 1: Table S2). Similarly, in fully adjusted independent models, antenatal maternal anaemia was not associated with the cerebellar white matter, cerebellar cortex, total cerebral cortex, total grey matter, or total cerebral white matter (Additional File 1: Table S3).

### Antenatal maternal haemoglobin concentration and total volumes for the corpus callosum and caudate nucleus

**Fig. 2:**
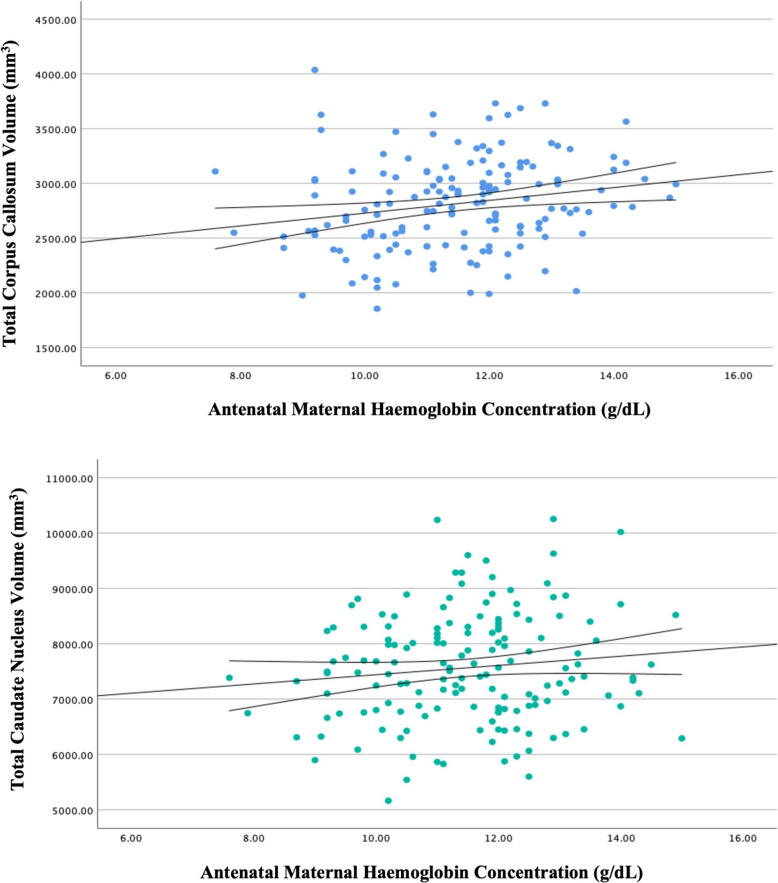
Linear regression of child total corpus callosum and caudate nucleus volumes by antenatal maternal haemoglobin concentration at 6–7 years of age. Unadjusted linear regression of maternal haemoglobin concentration (in grams per decilitre; to convert to grams per litre, multiply by 10) in pregnancy with child total corpus callosum (top) and total caudate nucleus (bottom) volumes (in millimetres cubed) with line of best fit and 95% confidence intervals

Given the demonstrated associations between antenatal maternal anaemia status and child brain volumes of the corpus callosum and bilateral caudate nuclei, these brain regions were explored further using multivariable linear regression for continuous haemoglobin concentrations (see Fig. 2).^1^

In a fully adjusted linear regression model, maternal haemoglobin concentration in pregnancy was positively associated with total corpus callosum volume (*β* = 0.239 [CI 0.10 to 0.38), *p* < 0.001). This suggests that an incremental increase of 1 g/dL in haemoglobin represents a 0.239 ${mm}^{3}$ increase in the volume of the corpus callosum. The overall model was significant (*F*[7,156] = 8.650, *p* < 0.001) and antenatal maternal haemoglobin accounted for 5.4% of the variance in total corpus callosum volume over and above covariates. The hierarchical inclusion of antenatal alcohol exposure (*β* = 0.115, *p* = 0.126) did not significantly contribute (*r*^*2*^ change = 0.019, *p* = 0.053) to the model.

Similarly, antenatal maternal haemoglobin concentration was positively associated with total caudate nucleus volume (*β* = 0.165 [CI 0.02 to 0.31], *p* = 0.027) in a fully adjusted linear regression model. Therefore, an incremental increase of 1 g/dL in haemoglobin represents a 0.165 ${mm}^{3}$ increase in the volume of the caudate nucleus. The overall model was significant (*F*[7,156] = 6.376, *p* < 0.001), and the addition of maternal haemoglobin concentration accounted for 2.6% of the variance in the total caudate nucleus volume when adjusting for covariates. In contrast, antenatal alcohol exposure (*β* = −0.16, *p* = 0.833) did not significantly contribute (*r*^*2*^ change = 0, *p* = 0.925) to the hierarchical model.

### Antenatal maternal anaemia status, postnatal child anaemia status, and child brain structure

In this neuroimaging study, child haemoglobin data was available on a sub-group of 89 children (*Mean* [*SD*] age of 76.06 [4.84] months; 49 [55.06%] male) who also had imaging data and maternal haemoglobin data. Overall, 216 measurements (Additional File 1: Table S1) were observed due to the inclusion of multiple measurements for the same child at different timepoints where relevant (median age [IQR] at haemoglobin measurement across all measurements: 11.23 [3.74–23.67] months). Of the 89 children, 42 (47.19%) were classified as anaemic at least once in the postnatal period using age-specific thresholds (Additional File 1: Table S1, Table S4). In this sub-group, 34/89 (38.20%) of the mothers were found to be anaemic during pregnancy, of which 16/34 (47%) had mild anaemia and 18/34 (53%) had moderate anaemia.

There were no group differences in sample characteristics between children with haemoglobin measurements and without across the sample (*n* = 157; Additional File 1: Table S5), or between children with and without anaemia in the sub-group (*n* = 89; Additional File 1: Table S6). In line with the full sample, there were no differences in sample characteristics in this sub-group between children born to mothers with and without anaemia during pregnancy (*n* = 89; Additional File 1: Table S7), other than antenatal alcohol exposure which was more prevalent in anaemic mothers as noted previously.

Overall, there was no association between antenatal maternal anaemia and postnatal child anaemia in this group (*n* = 89; Additional File 1: Table S4), *χ*^2^ = 0.799, *p* = 0.372. However, to investigate whether postnatal child anaemia was contributing to group differences in regional child brain volumes, it was included in previously established models as a covariate. In this sub-analysis (*n* = 89), the fully adjusted models conducted for the main analyses on maternal anaemia were replicated with the addition of postnatal child anaemia (see Table 3). Overall, the previously identified effects of antenatal maternal anaemia on child brain volumes remained robust with very similar effect sizes when adjusting for postnatal child anaemia. Furthermore, in this model, child anaemia status was not found to be associated with corpus callosum, caudate nucleus, or putamen volumes.

**Table 3:** Effects of antenatal maternal anaemia on regional child brain volumes of interest in sub-analysis adjusting for the role of child anaemia (*n* = 89)

Brain region	Mean volume (mm^3^)				Maternal anaemia status			
					Unadjusted for postnatal child anaemia^1^		Adjusted for postnatal child anaemia^2^	
	No maternal anaemia Mean (SD) (*n* = 55)	Maternal anaemia Mean (SD) (*n* = 34)	Adjusted mean volume difference (95% CI)^f^	Adjusted mean volume difference (%)^f^	*p*^d^	Partial *η*^2^ (95% CI)	*p*^d^	Partial *η*^2^ (95% CI)
**Corpus callosum**								
Posterior^a^	739.26 (107.10)	648.20 (134.77)	− 95.61 (− 148 to − 43.23)	− 12.93	< 0.001***^e^	0.144	< 0.001***^e^	0.142
Mid-Posterior^a^	382.55 (78.37)	324.26 (85.89)	− 55.84 (− 90.88 to − 20.80)	− 14.60	0.002**^e^	0.113	0.002**^e^	0.112
Central^a^	480.91 (101.31)	423.62 (77.37)	− 49.88 (− 88.81 to − 10.94)	− 10.37	0.008**^e^	0.083	0.013**^e^	0.075
Mid-Anterior^a^	472.37 (113.13)	412.09 (76.51)	− 58.55 (− 100.21 to − 16.89)	− 12.39	0.004**^e^	0.096	0.006**^e^	0.089
Anterior^a^	837.14 (128.33)	777.14 (120.12)	− 55.52 (− 108.98 to − 2.06)	− 6.63	0.038*^e^	0.052	0.042*^e^	0.051
Body^c^	1335.83 (231.65)	1159.97 (202.02)	− 164.27 (− 252.09 to − 76.44)	− 12.30	< 0.001***	0.155	< 0.001***	0.148
Total^c^	2912.24 (398.14)	2585.31 (400.71)	− 315.40 (− 477.29 to − 153.50)	− 10.83	< 0.001***	0.164	< 0.001***	0.158
**Subcortical regions**								
Caudate nucleus								
Left^b^	3843.44 (511.01)	3594.18 (445.86)	− 264.96 (− 471.19 to − 58.73)	− 6.89	0.011*^e^	0.077	0.012*^e^	0.076
Right^b^	4041.03 (539.29)	3764.97 (458.04)	− 286.84 (− 503.17 to − 70.52)	− 7.10	0.010*^e^	0.080	0.010*^e^	0.080
Putamen								
Left^b^	5199.72 (509.41)	5041.97 (571.69)	− 168.68 (− 393.08 to 55.72)	− 3.24	0.132	0.028	0.139	0.027
Right^b^	5231.66 (501.82)	5120.00 (552.04)	− 121.58 (− 333.61 to 90.45)	− 2.32	0.242	0.017	0.257	0.016

*Note. Corpus callosum body: Sum of mid-posterior, central, and mid-anterior regions. Total Corpus callosum: Sum of posterior, mid-posterior, central, mid-anterior, and anterior regions

^1^Partially adjusted model including antenatal maternal anaemia status, ICV, child age and sex at scan, SES (indicated by maternal education and household income), and antenatal alcohol exposure

^2^Fully adjusted model including antenatal maternal anaemia status, ICV, child age and sex at scan, SES (indicated by maternal education and household income), antenatal alcohol exposure, and postnatal child anaemia

^a,b^Fully adjusted MANOVA models run separately for the corpus callosum and caudate nucleus regions

^c^Fully adjusted ANOVA models run separately on the body and total corpus callosum summed volumes

^d^*p* values are adjusted for covariates but are not corrected for multiple comparisons

^e^Survived FDR correction for multiple comparisons. For the body and total corpus callosum where univariate analyses (ANOVAs) were run, multiple comparisons was not applicable

^f^The adjusted mean difference was calculated from the fully adjusted MANOVA models via post-hoc pairwise comparison using estimated marginal means. A negative mean difference and the corresponding percentage difference represent a smaller volume in children born to mothers with anaemia during pregnancy

*p is significant at < 0.05, **p is significant at < 0.01, ***p is significant at < 0.001

### Sensitivity analyses

All identified associations between antenatal maternal anaemia and regional brain volumes (corpus callosum and caudate nucleus) were robust, remaining significant in sensitivity analyses before and after adjusting for trimester of pregnancy, antenatal smoking exposure, and maternal HIV status. This was also true when using haemoglobin concentrations that had been corrected (Hb—0.3 g/dL) for mothers who smoke, with marginally stronger effect sizes [60].

## Discussion

### Principal findings and implications

In this neuroimaging sub-study, antenatal maternal anaemia was associated with smaller child brain volumes of the corpus callosum and caudate nucleus at 6–7 years of age. In contrasting the results from this DCHS research on school-age children (6–7 years old) with previous work from the same birth cohort on toddlers (2–3 years old) [4], the adjusted volume differences for ROIs were found to be comparable between timepoints. This was evident with consistently smaller volumes of the corpus callosum (7% at 6–7 years versus 8% at 2–3 years) and caudate nucleus (6% at 6–7 years versus 5% at 2–3 years). Furthermore, the nature and the strength of the relationship between maternal haemoglobin concentrations and child volumes for the total corpus callosum (standardised coefficient of 0.24 at both timepoints) and caudate nucleus (standardised coefficient of 0.17 at 6–7 years versus 0.15 at 2–3 years) remained similar. However, in exploratory analyses, no other emerging subcortical brain regions were associated with antenatal maternal anaemia. Similarly, as seen at 2–3 years of age, postnatal child anaemia was still not associated with regional brain volumes of the corpus callosum, caudate nucleus, or putamen at 6–7 years of age. Overall, these results indicate that the effects of antenatal maternal anaemia on child brain structure persist with age and remain regionally consistent over time. Given that significant brain growth occurs between 2–3 and 6–7 years of age, these results suggest that exposure to maternal anaemia in utero may have long-lasting implications for brain development across early life, with regional volume differences that remain consistent and do not resolve with age.

While the corpus callosum is a vulnerable structure that is sensitive to in many in-utero exposures [55, 61–63], the basal ganglia are iron-rich deep grey nuclei [35, 36] that may be disproportionately affected by iron deficiency anaemia [39]. Given the magnitude of the volume difference in affected regions, and the known role of the corpus callosum and caudate nucleus in neuropsychological functioning [28, 34, 40–42], these findings are likely to be clinically important. This is corroborated by recent systematic reviews suggesting a direct relationship between maternal anaemia and iron status during pregnancy, and poorer offspring performance across domains of cognition, motor function, language, memory, and behaviour on standardised testing [19, 20]. In parallel to the neuroimaging findings linking maternal anaemia to altered child brain structure in a sub-group of DCHS toddlers [4], previous research from the broader cohort has also demonstrated a strong link between maternal anaemia and poor developmental outcomes on the Bayley Scales of Infant and Toddler Development (BSID-III) at the same age [1]. Therefore, the persistent effects of maternal anaemia on volumes of key brain structures in this group of children at 6–7 years of age in this study suggests an increased risk of lasting cognitive difficulties that may become more evident at school age.

In this nested neuroimaging cohort, the prevalence of antenatal maternal anaemia was 32%. This is consistent with WHO estimates for African countries [6] and reports of stagnated progress in global efforts to reduce anaemia in LMICs [5]. In addition to suggesting the antenatal period as important for the timing of anaemia interventions, this study highlights the necessity of a multifactorial approach that addresses its complex aetiology. In the South African context, various other risk factors become relevant in considering the physiological mechanisms for iron deficiency anaemia. For example, HIV infection may have a negative impact on iron bioavailability due to increased sequestration within the context of inflammation [64–67]. Similarity, antenatal alcohol use may contribute to iron deficiency directly by limiting the intestinal absorption of iron and disrupting iron homeostasis [56, 57], and indirectly by negatively impacting nutritional choices [68]. In turn, antenatal tobacco exposure, which is highly overlapping with alcohol consumption during pregnancy [69, 70], is known to increase haemoglobin levels [49] resulting in the underestimation of functional anaemia in people who smoke. While all of these risk factors were prevalent in this cohort across groups, antenatal alcohol exposure was significantly more common in women who were anaemic during pregnancy suggesting that this interaction may be particularly important.

In addition to potential interactions between iron deficiency anaemia and risk factors such as maternal HIV and alcohol exposure, there is a well-known overlapping association with the corpus callosum as an associated outcome of interest. For example, this structure has also been widely implicated in research on antenatal alcohol exposure [55, 61] as well as HIV infection [62, 63]. After statistically accounting for these covariates in analyses, the association of maternal anaemia in pregnancy with smaller corpus callosum volumes was found to be robust. However, the inclusion of alcohol consumption as a covariate did weaken its effect. Given that in utero exposure to HIV, alcohol, and tobacco are highly prevalent and often comorbid in this cohort and other high-risk LMIC communities, an integrated approach to understanding and preventing anaemia is necessary. This is an important consideration in ongoing work to accelerate anaemia reduction using prevention and treatment strategies [5].

While the prevention of anaemia includes addressing food insecurity, managing disease, and mitigating psychosocial risk, current treatments are via simple interventions such as iron supplementation [5]. Given the growing body of research suggesting that postnatal iron supplementation may be less effective in improving long-term cognitive outcomes in children [71–73], these results re-emphasise the importance of considering the antenatal period as an important opportunity. According to WHO policy [74], iron and folic acid in pregnancy are recommended as per standard antenatal care practice but may be insufficient to combat the current burden of maternal anaemia in LMICs. This is due to a myriad of challenges including unclear aetiologies and imprecise strategies for intervention, dosage insufficiency, late presentation to clinics for antenatal care, limited public knowledge around the importance of nutrition and iron supplementation, unaffordability of nutritious iron-rich foods, and unclear directions for optimised supplementation use [64, 65, 75]. Future work should be focused on increased community engagement for understanding barriers to screening for anaemia and iron deficiency, and opportunities for context-specific strategizing for optimisation of anaemia interventions. The consistency of findings in the same key brain structures across early childhood in this study highlight these regions as a potential focus for outcomes in future research on intervention.

### Limitations

While this study is the first to demonstrate the persistent effects of antenatal maternal anaemia on child brain structure, it has limitations to consider. Firstly, given that this is an observational birth cohort study, causality can certainly be queried. However, the groups were comparable, covariates were included in models, and analyses were robust to sensitivity analyses. Furthermore, the findings remained consistent with previous results at an earlier age, a temporal association was demonstrated, and there was evidence for a biological gradient using continuous haemoglobin concentrations. Secondly, the neuroimaging sub-group was embedded within a high-risk community with multiple potential confounders including maternal HIV and alcohol use in pregnancy. Although this contributes to complicated interactions which deserve further exploration, it is an ecologically valid representation of many LMICs and should not limit the generalisability of findings. Thirdly, this study is limited in its ability to determine the exact nature and cause of maternal anaemia due to the reliance on point-of-care testing for haemoglobin. In using the most severe presentation of maternal anaemia represented by the lowest haemoglobin measure in pregnancy, we were able to demonstrate robust findings that held even with the inclusion of mild and potentially transient cases. However, data on the duration of maternal anaemia may have provided further insight into its impact on child brain development. Fourthly, this study is restricted in its ability to assess the role of postnatal child anaemia due to power limitations and selection bias. This is because child haemoglobin data was only available for half the sample in a sub-group of children who had blood tests while ill with lower respiratory tract infections. Furthermore, the child anaemia variable was a dichotomous indication of whether a child had ever been diagnosed with anaemia which prevented the exploration of severity, duration, and age-specific associations. Lastly, due to power limitations, this study was unable to assess whether altered child brain structure in key affected ROIs mediated the relationship between antenatal maternal anaemia and school readiness. Future work should investigate longitudinal analyses with additional timepoints to assess how antenatal maternal anaemia affects trajectories of brain development, with the inclusion of comprehensive anaemia data, iron metrics, and cognitive outcomes.

## Conclusions

This nested neuroimaging cohort study demonstrated that associations of maternal anaemia in pregnancy with child brain volumes are regionally consistent and persist from age 2–3 years through to age 6–7 years. Overall, antenatal maternal anaemia was associated with smaller volumes of the corpus callosum and caudate nucleus in school-age children, with comparable adjusted volume differences and coefficients to findings in toddlers. The persistent associations of antenatal maternal anaemia with structural child brain findings in regions underlying important cognitive functions emphasises the need for optimised anaemia interventions in women of reproductive age before and during pregnancy for improved child neurodevelopmental outcomes. Given the high prevalence of comorbid antenatal alcohol consumption in anaemic women from this cohort, this risk factor is likely to play a key causal role via a physiological interaction with iron and nutritional behaviours. Therefore, prevention and intervention strategies for maternal anaemia should be multifaceted to account for overlapping risk factors such as malnutrition and alcohol use in pregnancy. Given the consistency of findings in key brain structures across early childhood, the importance of these regions is emphasised as a focus for future research, particularly on intervention outcomes.

## Supplementary Information

Supplementary Material 1: Additional File 1.

## Data Availability

The de-identified data that support the findings of this study are available upon reasonable request from the corresponding author as per Drakenstein Child Health Study (DCHS) cohort guidelines.

## Abbreviations

ADHD: Attention-deficit hyperactivity disorder
ANOVA: Analysis of variance
ASD: Autism spectrum disorder
ASSIST: Alcohol, Smoking, and Substance Involvement Screening Test
BMI: Body mass index
BMIZ: Body mass index-for-age
CI: Confidence interval
CUBIC: Cape Universities Body Imaging Centre
DCHS: Drakenstein Child Health Study
FDR: False discovery rate
GLM: general linear model
HAZ: Height-for-age
Hb: Haemoglobin
HCZ: Head circumference-for-age
HIV: Human immunodeficiency virus
ICV: Intracranial volume
LMIC: Low- and middle-income country
MANOVA: Multivariate analysis of variance
MRI: Magnetic resonance imaging
QC: Quality control
ROI: Region-of-interest
SD: Standard deviation
SDG: Sustainable Development Goal
SES: Socio-economic status
SI: Supplementary information
SPSS: Statistical Package for the Social Sciences
WAZ: Weight-for-age
WHO: World Health Organization
ZAR: South African Rand

## Footnotes

1 In assessing the association between antenatal maternal haemoglobin concentrations and the corpus callosum, we focused on the total volume as it demonstrated the largest effect. Similarly, given that antenatal maternal anaemia status was associated with effect on the caudate nucleus bilaterally, the total volume was entered into the regression model for this region.
