# Supplementary material for: Persistent impact of antenatal maternal anaemia on child brain structure at 6–7 years of age: a South African child health study
Source: BMC Med. 2025 Feb 21;23:94. doi: 10.1186/s12916-024-03838-6 (PMC11846184; doi:10.1186/s12916-024-03838-6)
Supplement: Supplementary file 1 — Supplementary Material 1: Additional File 1. [file 12916_2024_3838_MOESM1_ESM.pdf]

Persistent Impact of Antenatal Maternal Anaemia on Child Brain Structure at 6-7 Years of Age: A South African Child Health Study

SUPPLEMENTARY INFORMATION

Figure S1. Drakenstein Child Health Study Flowchart for Neuroimaging at 6-7 Years

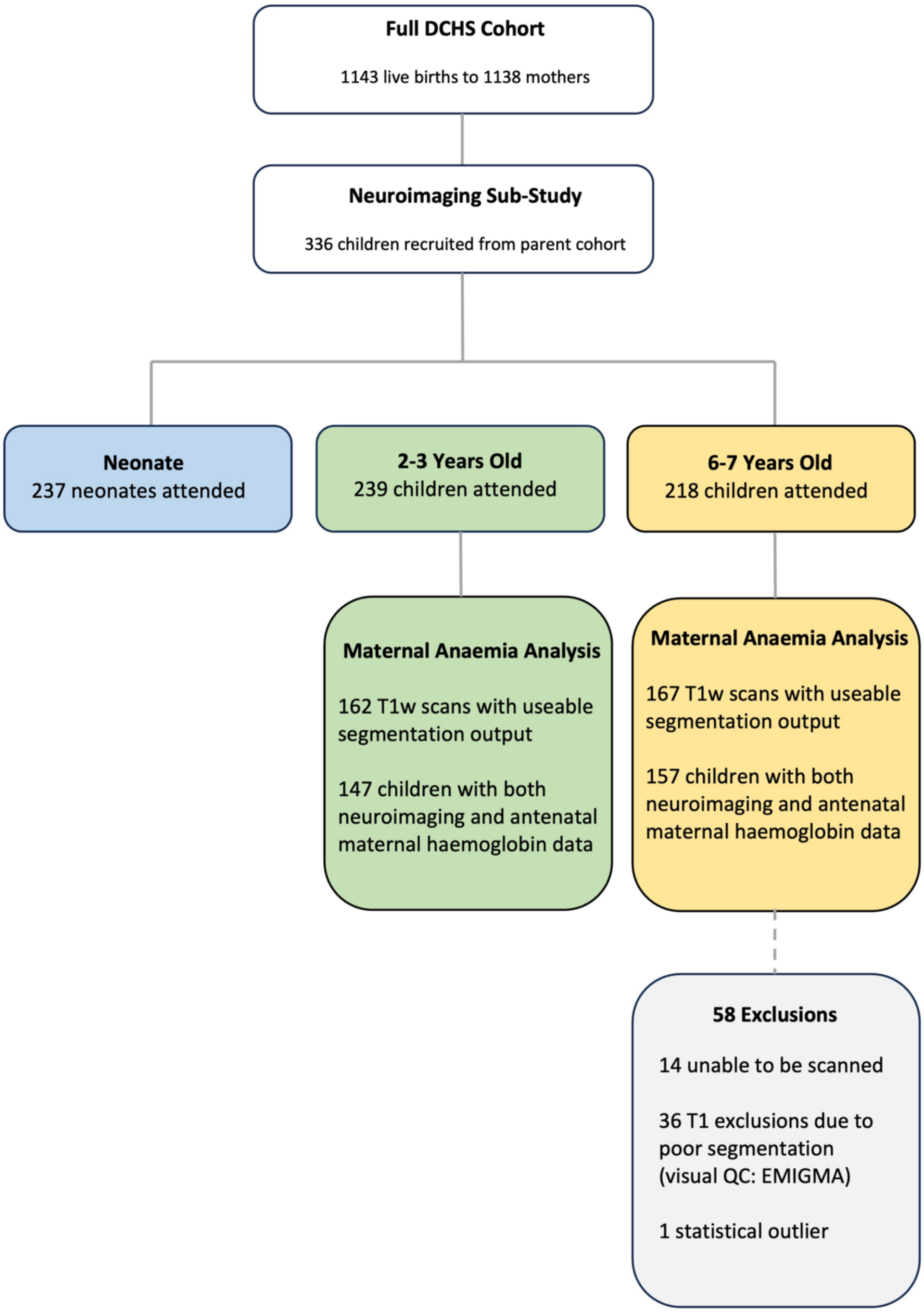

Figure S1 Footnote. The full details for the Drakenstein Child Health Study (DCHS) cohort as well as the anaemia analysis results from the 2-3-year timepoint have been published previously. Exclusions of acquired data at 6-7 years were based on a rigorous quality check conducted by two senior researchers following the ENIGMA protocol.

Table S1. Classification of Postnatal Child Anaemia by Age

Child Age at Haemoglobin Measurement	Haemoglobin Concentration Threshold for Anaemia[a]	No. (%) of Haemoglobin Measurements Observed (*n* = 216)[b]	No. (%) of Aneamic Observations (*n* = 80)	No. (%) of Non-Anaemic Observations (*n* = 136)
0 – 3 days	<14g/dL	8 (3.70)	1 (2.25)	7 (5.15)
3 days – 1 month	<15g/dL	1 (0.46)	1 (1.25)	0 (0)
1 – 2 months	<11.5g/dL	15 (6.94)	9 (11.25)	6 (4.41)
2 - 3 months	<9.4g/dL	16 (7.41)	5 (6.25)	11 (8.09)
3 months – 6 months	<11.1g/dL	39 (18.01)	22 (27.50)	17 (12.50)
6 months to 60 months	<11.0g/dL	136 (62.96)	42 (52.50)	94 (69.12)
60 months +	<11.5	1 (0.46)	0 (0)	1 (0.74)

[a]Reference ranges for children between 0 and 6 months were obtained from GSH/UCT Pathology Laboratory guidelines, Groote Schuur Hospital, National Health Laboratory Service (Western Cape); effective date 23 January 2003. WHO guidelines were used for children over 6 months.
[b]Observations represent all child haemoglobin measurements, including multiple measurements for the same child at different timepoints. In a sample of 89 children with haemoglobin data, 216 measurements were observed.

Text S1. Drakenstein Child Health Study MRI Protocol at the 6-7 Year Timepoint

3D Magnetization Prepared Rapid Acquisition Gradient Echo (MPRAGE) were acquired on a 3T Siemens Skyra MRI system in the sagittal orientation with the following parameters: repetition time=2500ms; echo time= 3.35ms; flip angle=8.0°; voxel size 1.0x1.0x1.0mm^3; inversion time=1000ms; field of view=256mm; 176 slices, 1.0mm thick. Scan time: 7min12s. Freesurfer segmentation output was subject to rigorous quality control protocol by two independent senior researchers using the ENIGMA pipeline (http://enigma.ini.usc.edu/protocols/imaging-protocols/). All scans were reviewed and reported on by a radiologist. Any qualitative abnormalities or incidental findings were discussed with a paediatric neurologist and, where appropriate, referred via established clinical pathways. These scans were excluded from analysis.

Table S2. Exploratory Analysis of Subcortical Brain Regions by Antenatal Maternal Anaemia Status (*n* = 157)

Subcortical Brain Region	Mean Volume (mm^3)				Maternal Anaemia Status Fully Adjusted Model[a]	
	No Maternal Anaemia Mean (SD) (*n* = 107)	Maternal Anaemia Mean (SD) (*n* = 50)	Adjusted Mean Volume Difference (95% CI)[b]	Adjusted Mean Volume Difference (%)[b]	*p*	*Partial* η^2 *(95% CI)*
Amygdala						
Left	1409.07 (191.16)	1410.82 (189.03)	-21.46 (-75.20 – 32.27)	-1.52	.431	.004
Right	1552.28 (195.01)	1561.72 (205.41)	-14.18 (-70.37 – 42.01)	-0.91	.619	.002
Hippocampus						
Left	3520.02 (301.19)	3505.01 (360.78)	-35.34 (-134.27 – 63.59)	-1.00	.481	.003
Right	3604.78 (348.81)	3597.65 (353.91)	-23.62 (-124.85 – 77.60)	-0.66	.645	.001
Thalamus						
Left	7244.23 (649.90)	7118.72 (705.98)	-89.33 (-311.21 – 132.55)	-1.23	.428	.004
Right	7085.08 (631.03)	6959.24 (620.79)	-107.59 (-313.72 – 98.54)	-1.52	.304	.007
Nucleus Accumbens						
Left	659.45 (100.74)	678.38 (112.68)	12.85 (-20.49 – 46.20)	1.95	.447	.004
Right	659.74 (95.44)	683.93 (95.46)	18.31 (-11.54 – 48.17)	2.78	.227	.010
Pallidum						
Left	1817.48 (202.63)	1775.26 (226.18)	-41.14 (-103.95 – 21.68)	-2.26	.198	.011
Right	1729.04 (211.04)	1671.04 (254.58)	-58.75 (-127.63 – 10.12)	-3.40	.094	.019

[a]Fully adjusted model including antenatal maternal anaemia status, ICV, child age and sex at scan, SES (indicated by maternal education and household income), and antenatal alcohol exposure.

[b]The adjusted mean difference was calculated from the fully adjusted MANOVA models via post-hoc pairwise comparison using estimated marginal means. A negative mean difference and the corresponding percentage difference represent a smaller volume in children born to mothers with anaemia during pregnancy.

Table S3. Exploratory Analysis of Total Cerebral Volumes and The Cerebellum by Antenatal Maternal Anaemia Status (*n* = 157)

Brain Region	Mean Volume (mm³)				Maternal Anaemia Status Fully Adjusted Model[a]	
	No Maternal Anaemia Mean (SD) (*n* = 107)	Maternal Anaemia Mean (SD) (*n* = 50)	Adjusted Mean Volume Difference (95% CI)[b]	Adjusted Mean Volume Difference (%)[b]	*p*	*Partial η²* *(95% CI)*
Cerebellar White Matter						
Left	12313.49 (1654.82)	11909.82 (1441.48)	-410.64 (-921.83 – 100.55)	-3.33	0.115	0.017
Right	11502.51 (1517.33)	11294.14 (1352.42)	-259.66 (-707.59 – 188.26)	-2.26	0.254	0.009
Cerebellar Cortex						
Left	54789.02 (5696.79)	54063.82 (5158.82)	-856.10 (-2457.04 – 744.84)	-1.56	0.292	0.007
Right	54419.22 (5860.09)	53573.85 (5272.61)	-1056.48 (-2688.37 – 575.40)	-1.94	0.203	0.001
Total Grey Matter	718678.81 (52822.82)	713612.20 (54289.65)	-5258.02 (-18350.29 – 7834.26)	-0.73	0.429	0.004
Total Cerebral Cortex						
Left	277322.78 (21662.36)	276204.55 (21565.99)	-887.39 (-6604.68 – 4829.90)	-0.32	0.759	0.001
Right	276450.56 (22412.60)	275140.87 (22132.00)	-1264.38 (-7164.45 – 4635.68)	-0.46	0.673	0.001
Total Cerebral White Matter						
Left	178090.95 (20303.72)	178908.13 (21170.43)	-412.59 (-4390.38 – 5215.56)	-0.23	0.865	0.000
Right	178065.01 (20737.35)	178870.93 (21078.28)	-209.44 (-4710.37 – 5129.26)	-0.12	0.933	0.000

[a]Fully adjusted model including antenatal maternal anaemia status, ICV, child age and sex at scan, SES (indicated by maternal education and household income), and antenatal alcohol exposure. Separate models were run for cerebellar white matter, cerebellar cortex, total grey matter, total cerebral cortex, and total cerebral white matter.
[b]The adjusted mean difference was calculated from the fully adjusted MANOVA models via post-hoc pairwise comparison using estimated marginal means. A negative mean difference and the corresponding percentage difference represent a smaller volume in children born to mothers with anaemia during pregnancy.

Table S4. Number of Children With Both Maternal and Child Haemoglobin Measurements (*n* = 89)

Antenatal Maternal Anaemia	Postnatal Child Anaemia		Total
	Yes *n* (%)	No *n* (%)	
**Yes**	14 (41.18)	20 (58.82)	34
**No**	28 (50.91)	27 (49.01)	55
**Total**	42	47	89

Chi-squared test for association between antenatal maternal anaemia and postnatal child anaemia, $p = 0.372$.

Table S5. Sample Characteristics of Children With and Without Haemoglobin Data (*n* = 157)

Variable[a]	Total Sample (*n* = 157)		
	Child Haemoglobin Measurements (*n* = 89)	No Child Haemoglobin Measurements (*n* = 68)	*p*
**Maternal Characteristics**			
Monthly household income (ZAR)			
<1000	28 (31.46)	17 (25)	
1000-5000	52 (58.43)	40 (58.82)	0.433
>5000	9 (10.11)	11 (16.18)	
Education[b]			
Primary	8 (8.99)	3 (4.41)	
Some secondary	48 (53.93)	35 (51.47)	0.568
Completed secondary	29 (32.58)	28 (41.18)	
Tertiary	4 (4.49)	2 (2.94)	
Employment (Employed)	23 (25.84)	26 (38.24)	0.097
Age at delivery (years)	27.64 (5.66)	27.84 (6.04)	0.833
Smoking during pregnancy	32 (35.96)	23 (33.82)	0.781
Alcohol during pregnancy	28 (31.46)	20 (29.41)	0.782
HIV infection during pregnancy	29 (32.58)	20 (29.41)	0.671
Weight 6 weeks postpartum (kg)[c]	71.28 (17.84)	67.04 (14.18)	0.167
BMI 6 weeks postpartum (kg)[c]	28.13 (6.62)	26.82 (5.04)	0.244
**Child Characteristics**			
Age at scan (months)	76.06 (4.84)	74.87 (4.61)	0.122
Sex (boys)	49 (55.01)	35 (51.47)	0.655
HIV infection	0	0	n/a
Gestational age at birth (weeks)	38.66 (2.47)	39.07 (1.73)	0.244
Birth weight (g)[d]	3071.82 (672.94)	3080.00 (501.26)	0.933
Birth length (cm)[d]	49.16 (4.13)	49.71 (3.36)	0.367
Birth head circumference (cm)[d]	33.46 (2.31)	33.65 (1.71)	0.568
WAZ at 6 years[c,d]	-0.23 (1.12)	-0.37 (1.02)	0.410
HAZ at 6 years[c,d]	-0.32 (0.99)	-0.39 (1.14)	0.699
BMIZ at 6 years[c,d]	-0.07 (1.03)	-0.24 (1.00)	0.299
**Child Neuroanatomical Variables**			
Intracranial volume (mm^3)	1322768.18 (153678.75)	1300173.97 (130432.07)	0.332

Abbreviations: Hb, haemoglobin; BMI, body mass index (calculated as weight in kilograms divided by height in meters squared); g, grams; HAZ, z-scores for height-for-age; BMIZ, z-scores for BMI-for-age; WAZ, z-scores for weight-for-age; ZAR, South African Rand.

SI conversion factor: To convert to haemoglobin grams per litre, multiply by 10.

[a]Values for continuous variables are presented as: mean ± standard deviation (range). Values for categorical variables are presented as: number (%).

[b] Fisher's exact test result interpreted due to one or more cells having an expected count of less than 5.

[c] Missing values: maternal weight 6 weeks postpartum (*n*=40), maternal BMI 6 weeks postpartum (*n*=41), WAZ at 6 years (*n*=2), HAZ at 6 years (*n*=2), BMIZ at 6 years (*n*=3).

[d]The birth anthropometric measurements were conducted by trained labour staff in the ward. Infant length was measures in cm to the nearest completed 0.5cm and weight was measured in kgs (and converted to grams). Child weight and length measurements at 6-7 years of age were converted to z-scores based on age and sex using Anthro software for WAZ, HAZ, and HCZ. Children were classified as underweight, stunted, or having microcephaly if they had z-scores of less than -2 SDs.

**p* is significant at <0.05, ***p* is significant at <0.01, ****p* is significant at <0.001.

Table S6. Sample Characteristics of Children With and Without Postnatal Anaemia (*n* = 89)

Variable[a]	Total Sample (*n* = 89)		
	Child Anaemia (*n* = 42)[b]	No Child Anaemia (*n* = 47)[b]	*p*
**Maternal Characteristics**			
Monthly household income (ZAR)[c]			
<1000	16 (38.10)	12 (25.53)	0.397
1000-5000	23 (54.76)	29 (61.70)	
>5000	3 (7.14)	6 (12.77)	
Education[c]			
Primary	4 (9.52)	4 (8.51)	0.895
Some secondary	24 (57.14)	24 (51.06)	
Completed secondary	12 (28.57)	17 (36.17)	
Tertiary	2 (4.76)	2 (4.26)	
Employment	11 (26.19)	12 (25.53)	0.944
Age at delivery (years)	27.67 (5.89)	27.62 (5.52)	0.967
Smoking during pregnancy	16 (38.10)	16 (34.04)	0.691
Alcohol during pregnancy	12 (28.57)	16 (34.04)	0.579
HIV infection during pregnancy	15 (35.71)	14 (29.79)	0.551
Weight 6 weeks postpartum (kg)[d]	74.55 (19.80)	68.01 (15.24)	0.137
BMI 6 weeks postpartum (kg)[d]	29.33 (7.32)	26.97 (5.75)	0.153
**Child Characteristics**			
Age at scan (months)	75.88 (5.12)	76.21 (4.63)	0.749
Sex (boys)	23 (54.76)	26 (55.32)	0.958
HIV infection	0	0	n/a
Gestational age at birth (weeks)[f]	38.69 (1.66)	38.64 (3.03)	0.919
Birth weight (g)[e]	3128.38 (608.25)	3021.28 (728.72)	0.457
Birth length (cm)[e]	49.31 (4.05)	49.02 (4.24)	0.744
Birth head circumference (cm)[e,f]	33.80 (1.73)	33.15 (2.71)	0.179
WAZ at 6 years[d,e]	-0.01 (1.10)	-0.42 (1.11)	0.088
HAZ at 6 years[d,e]	-0.21 (0.91)	-0.42 (1.06)	0.313
BMIZ at 6 years[d,e]	0.15 (1.13)	-0.26 (0.89)	0.069
**Child Neuroanatomical Variables**			
Intracranial volume (mm^3)	1344589.07 (165001.18)	1303268.66 (141743.78)	0.207

Abbreviations: Hb, haemoglobin; BMI, body mass index (calculated as weight in kilograms divided by height in meters squared); g, grams; HAZ, z-scores for height-for-age; BMIZ, z-scores for BMI-for-age; WAZ, z-scores for weight-for-age; ZAR, South African Rand.

SI conversion factor: To convert to haemoglobin grams per litre, multiply by 10.

[a]Values for continuous variables are presented as: mean ± standard deviation (range). Values for categorical variables are presented as: number (%).

[b] Child anaemia classified using age-specific thresholds (see Table S1).

[c]Fisher's exact test result interpreted due to one or more cells having an expected count of less than 5.

[d]Missing values: maternal weight 6 weeks postpartum (*n*=23), maternal BMI 6 weeks postpartum (*n*=24), WAZ at 6 years (*n*=2), HAZ at 6 years (*n*=1), BMIZ at 6 years (*n*=2).

[e]The birth anthropometric measurements were conducted by trained labour staff in the ward. Infant length was measures in cm to the nearest completed 0.5cm and weight was measured in kgs (and converted to grams). Child weight and length measurements at 6-7 years of age were converted to z-scores based on age and sex using Anthro software for WAZ, HAZ, and HCZ. Children were classified as underweight, stunted, or having microcephaly if they had z-scores of less than -2 SDs.

[f]Levene's test was significant. T-test results were interpreted based on equal variance not assumed

**p* is significant at <0.05, ***p* is significant at <0.01, ****p* is significant at <0.001.

Table S7. Sample Characteristics of Children Born to Mothers With and Without Anaemia During Pregnancy in Sub-Group with Postnatal Child Anaemia Data (*n* = 89)

Variable[a]	Total sample (*n* = 89)		
	Maternal Anaemia (*n* = 34)	No Maternal Anaemia (*n* = 55)	*p*
**Pregnancy Characteristics**			
Anaemia status in pregnancy[b]			
Mild	16 (47.06)	n/a	n/a
Moderate	18 (52.94)	n/a	n/a
Severe	0	n/a	n/a
Maternal Hb during pregnancy (g/dL)[e]	9.76 (0.68)	12.21 (0.96)	<0.001***
Trimester of pregnancy Hb measured[c,f]			
First	12 (35.29)	35 (63.63)	
Second	21 (61.76)	20 (36.36)	0.012*
Third	1	0	
**Maternal Characteristics**			
Monthly household income (ZAR)[f]			
<1000	10 (29.41)	18 (32.73)	
1000-5000	21 (61.76)	31 (56.36)	0.948
>5000	3 (8.82)	6 (10.91)	
Education[f]			
Primary	4 (11.76)	4 (7.27)	
Some secondary	19 (55.88)	29 (52.73)	0.828
Completed Secondary	10 (29.41)	19 (34.55)	
Tertiary	1 (2.94)	3 (5.45)	
Employed	8 (23.53)	15 (27.27)	0.695
Age at delivery (years)	28.03 (6.01)	27.40 (5.48)	0.613
Smoking during pregnancy	11 (32.35)	21 (38.18)	0.578
Alcohol during pregnancy	16 (47.06)	12 (21.82)	0.013*
HIV infection during pregnancy	13 (38.24)	16 (29.10)	0.371
Weight 6 weeks postpartum (kg)[g]	68.74 (19.94)	72.55 (16.79)	0.417
BMI 6 weeks postpartum (kg)[g]	27.26 (7.41)	28.58 (6.22)	0.449
**Child Characteristics[d]**			
Age at scan (months)	76.00 (5.14)	76.09 (4.70)	0.932
Sex (boys)	18 (52.94)	31 (56.36)	0.752
HIV infection	0	0	n/a
Gestational age at birth (weeks)	38.32 (2.88)	38.87 (2.18)	0.310
Birth weight (g)[d]	3095.29 (736.52)	3057.31 (637.12)	0.798
Birth length (cm)[d]	48.94 (4.15)	49.29 (4.15)	0.700
Birth head circumference (cm)[d]	33.55 (2.62)	33.40 (2.11)	0.758
WAZ at 6 years[d,g]	-0.42 (1.28)	-0.11 (1.00)	0.208
HAZ at 6 years[d,g]	-0.48 (1.14)	-0.22 (0.89)	0.230
BMIZ at 6 years[d,g]	-0.19 (1.00)	0.01 (1.04)	0.369
**Child Neuroanatomical Variables**			
Intracranial volume (mm^3)	1333004.12 (134554.96)	1316440.51 (165276.38)	0.624

Abbreviations: Hb, haemoglobin; BMI, body mass index (calculated as weight in kilograms divided by height in meters squared); g, grams; HAZ, z-scores for height-for-age; BMIZ, z-scores for BMI-for-age; WAZ, z-scores for weight-for-age; ZAR, South African Rand.

SI conversion factor: To convert to haemoglobin grams per litre, multiply by 10.

[a]Values for continuous variables are presented as: mean ± standard deviation (range). Values for categorical variables are presented as: number (%).

[b]Maternal anaemia during pregnancy was classified according to the WHO threshold of Hb<11g/dL. Severity classifications were defined as mild (10.0-10.9g/dL), moderate (7.0-9.9g/dL), and severe (<7.0g/dL).

[c]Trimester of pregnancy defined as first (0-12 weeks), second (13-27 weeks), and third (28 weeks onwards).

[d]The birth anthropometric measurements were conducted by trained labour staff in the ward. Infant length was measures in cm to the nearest completed 0.5cm and weight was measured in kgs (and converted to grams). Child weight and length measurements at 6-7 years of age were converted to z-scores based on age and sex using Anthro software for WAZ, HAZ, and HCZ. Children were classified as underweight, stunted, or having microcephaly if they had z-scores of less than -2 SDs.

[e]Levene's test was significant. T-test results were interpreted based on equal variance not assumed.

[f]Fisher's exact test result interpreted due to one or more cells having an expected count of less than 5.

[g]Missing values: maternal weight 6 weeks postpartum (*n*=23), maternal BMI 6 weeks postpartum (*n*=24), WAZ at 6 years (*n*=2), HAZ at 6 years (*n*=1), BMIZ at 6 years (*n*=2).

**p* is significant at <0.05, ***p* is significant at <0.01, ****p* is significant at <0.001.
